# Lack of Association of a Functional Polymorphism in the Serotonin Receptor Gene With Body Mass Index and Depressive Symptoms in a Large Meta-Analysis of Population Based Studies

**DOI:** 10.3389/fgene.2018.00423

**Published:** 2018-10-02

**Authors:** Beverly H. Brummett, Michael A. Babyak, Abanish Singh, Elizabeth R. Hauser, Rong Jiang, Kim M. Huffman, William E. Kraus, Svati H. Shah, Ilene C. Siegler, Redford B. Williams

**Affiliations:** ^1^Department of Psychiatry and Behavioral Sciences, Duke University Medical Center, Durham, NC, United States; ^2^Duke Molecular Physiology Institute, Duke University Medical Center, Durham, NC, United States; ^3^Department of Biostatistics and Bioinformatics, Duke University Medical Center, Durham, NC, United States; ^4^Cooperative Studies Program Epidemiology Center – Durham Veterans Affairs Medical Center, Durham, NC, United States; ^5^Division of Cardiology, Duke University School of Medicine, Durham, NC, United States

**Keywords:** rs6318, depressive symptoms, body mass index, stress, sex, race

## Abstract

The serotonin receptor 5-HTR2C is thought to be involved in the function of multiple brain structures. Consequently, the *HTR2C* gene has been studied extensively with respect to its association with a variety of phenotypes. One coding variant in the *HTR2C* gene, Cys23Ser (rs6318), has been associated with depressive symptoms. and adiposity; however, these findings have been inconsistent. The reasons for this mixed picture may be due to low statistical power or due to other factors such as failure to account for possible interacting environmental factors, such as psychosocial stress. Further, the literature around this polymorphism is marked by limited inclusion of persons of African ancestry. The present study sought to overcome these limitations and definitively determine the relationship of this polymorphism with depressive and obesity phenotypes in a large sample meta-analysis. Thus, we harmonized individual level data from 10 studies including the Women’s Health Initiative, CARDIA, ARIC, Framingham Offspring, and the Jackson Heart Study, resulting in a sample of 27,161 individuals (10,457 Black women, 2,819 Black men, 7,419 White women, and 6,466 White men). We conducted a random effects meta-analysis using individual level data to examine whether the Cys23Ser variant—either directly, or conditionally depending on the level of psychosocial stress—was associated with depressive symptoms and body mass index (BMI). We found that psychosocial stress was associated with both depression and BMI, but that Cys23Ser was not directly associated with, nor did it modify the associations of psychosocial stress with depression or BMI. Thus, in the largest study of this polymorphism, we have determined that rs6318 is not associated with depression, or BMI.

## Introduction

The 5-HTR2C serotonin receptor plays critical roles in numerous human neural circuits (Drago and Serretti, 2009) and is coded by the X chromosome gene *HTR2C.* Variation in the *HTR2C* gene has been associated with a wide variety of phenotypes: psychological disorders, feeding behavior, antipsychotic medication-induced side effects, clinical response to antidepressant medications, stress-induced mesoaccumbal dopamine release sensitivity (Drago and Serretti, 2009), pathogenesis of major affective disorders (Yildirim and Derksen, 2013), cortisol response to stress (Brummett et al., 2013; Brummett et al., 2014a; Avery and Vrshek-Schallhorn, 2016; Way et al., 2016), adiposity (Westberg et al., 2002; Hu et al., 2003), and risk for incident cardiovascular events (Brummett et al., 2013). The common *HTR2C* Cys23Ser (rs6318) variant—a non-synonymous coding single nucleotide polymorphism (SNP)—has been the focus of much of this investigation. This biallelic SNP is likely functional, with the Ser23 C allele having been shown to be constitutively more active than the Cys23 G allele (Okada et al., 2004). It has been associated with differences in blood flow in areas of the brain associated with emotional response, such as the anterior cingulate cortex (Drago and Serretti, 2009), and with variation of cerebral spinal fluid monoamine levels (Lappalainen et al., 1999). Rs6318 also has been associated with two important psychological and biological phenotypes—depressive symptoms (Lerer et al., 2001; Drago and Serretti, 2009; Brummett et al., 2014b) and adiposity (Westberg et al., 2002; Hu et al., 2003; Praschak-Rieder et al., 2005; Drago and Serretti, 2009). In our own work, this SNP was associated with multiple phenotypes including depressive symptoms, cortisol response to stress, and risk of incident cardiovascular events. An in-depth discussion of the role of the 5-HTR2C serotonin receptor and the rs6318 SNP appears in Drago and Serretti (2009).

Despite the relatively extensive background suggesting the role of this functional SNP with brain and cardiometabolic diseases, there have been inconsistencies in the observed relationships between rs6318 and those phenotypes. Further, variation in this SNP has not been evaluated in adequate sample sizes of African Americans, nor has the role of brain and heart related environmental factors as effect modifiers been evaluated sufficiently. We attempted to address these challenges by conducting the largest analysis to date of rs6318 by using a large harmonized dataset from 10 population based studies, (four from studies conducted at Duke and six from publicly available dbGaP data (Singh et al., 2018), for a total of 27,161 individuals, of whom 13,276 were self-identified as Black. This large data set afforded the possibility of generating estimates of association that were more likely to be robust to sample instability, and also to more fully examine these associations including men and women of both European and African ancestry. In addition to confirming prior associations between rs6318 with depression and obesity, we hypothesized the presence of a gene-environment interaction between rs6318, chronic psychosocial, stress, and gender.

## Materials and Methods

### Study Populations

We used ten datasets in this study, including 6 large public-access datasets and four Duke University Medical Center (DUMC) datasets. Each of the public-access datasets were obtained from the data depository dbGaP/database of Genotypes and Phenotypes/National Center for Biotechnology Information, National Library of Medicine (NCBI/NLM)/ https://www.ncbi.nlm.nih.gov/gap (Mailman et al., 2007) through an authorized access. Below is a brief description of all the contributing studies. All subjects, in each study, gave written informed consent in accordance with the Declaration of Helsinki.

#### The Women’s Health Initiative (WHI)

The Women’s Health Initiative (WHI) is a long-term national health study dedicated to developing prevention strategies for heart disease, breast and colorectal cancer, and osteoporotic fractures in postmenopausal women (The WHI Study Group, 1998). The available data from this study included only Black and Hispanic participants. Our analysis uses data from only the Black participants from this dataset.

#### The Coronary Artery Risk Development in Young Adults Study (CARDIA)

The Coronary Artery Risk Development in Young Adults Study (CARDIA) was designed to study the etiology and natural history of cardiovascular disease beginning in young adulthood (Friedman et al., 1988). CARDIA contains roughly equal numbers of individuals in the subgroups of race, gender, and education.

#### Atherosclerosis Risk in Communities Study (ARIC)

Atherosclerosis Risk in Communities Study (ARIC) is a prospective epidemiologic study focused to investigate the etiology and natural history of atherosclerosis and demographic variation in cardiovascular risk factors, medical care, and disease (The ARIC Study Group Investigators, 1989). The study examined atherosclerosis by direct observation and by use of modern biochemistry. The components of the study included identification, investigation, and diagnosis of clinical events through home interviews, clinic examinations, and annual telephone follow-ups.

#### Framingham Offspring Cohort

We used the Generation 2 (or Offspring) dataset from the Framingham Heart Study Cohort for this work (Feinleib et al., 1975) because of availability of psychosocial measurements and genetic data. The second-generation cohort included adult children (and their spouses) of the original participants. The cohort is almost entirely White; we elected therefore to use only White participants from this dataset. Because the sample included related participants, we maintained only a single participant (the case with the lowest ID number) from each family cluster.

#### Multi-Ethnic Study of Atherosclerosis (MESA)

Multi-Ethnic Study of Atherosclerosis (MESA) was designed to study cardiovascular disease (CVD) risk factors that predict progression of the clinically observable or subclinical cardiovascular disease; (Bild et al., 2002).

#### Jackson Heart Study (JHS)

The Jackson Heart Study (JHS) is a large, community-based, observational study that was designed to explore reasons for the prevalence of cardiovascular disease among African Americans (Sempos et al., 1999). The study participants were recruited from urban and rural areas of the Jackson, MS metropolitan statistical area (MSA).

#### Community Health and Stress Evaluation (CHASE) Study

The DUMC CHASE Study was designed to determine the role of psychosocial and biobehavioral factors in the etiology of CVD. The study included a lumbar puncture procedure along with medical and psychosocial information survey of Black and White participants from a wide range of socioeconomic status (Burroughs et al., 2003).

#### Studies of a Targeted Risk Reduction Intervention Through Defined Exercise (ST**RRID**E)

We used baseline data from two of the three DUMC ST**RRID**E clinical trials: ST**RRID**E – Aerobic Training / Resistance Training (AT/RT), and ST**RRID**E pre-diabetes (PD). ST**RRID**E AT/RT study was designed to compare the effects of aerobic training (AT) and resistance training (RT) and the full combination (AT/RT) on central ectopic fat and liver enzymes and fasting insulin resistance by homeostatic model assessment (HOMA) (Slentz et al., 2011). The purpose of the ST**RRID**E-PD study was to compare the effects of different amounts and intensities of exercise training programs without diet to an exercise and diet program modeled after the first six months of the Diabetes Prevention Program (DPP) (Slentz et al., 2016). During the course of analysis, we observed that in both Black and White men in the ST**RRID**E AT/RT study there was insufficient variability in the stress measure to model the stress by SNP interaction. Thus, data from this study was excluded from the analysis of males.

#### Duke Caregiver Study (DCS)

This DUMC study included data from family caregivers of a relative with Alzheimer’s disease or other dementia and a non-caregiving comparison group (Siegler et al., 2010). The study was designed to examine the interaction of stress and genetic markers as predictors of CVD.

#### Duke Family Heart Study (DFHS)

This DUMC study was designed to examine the effect of genetic variation on the relationship between psychosocial and cardiovascular risk factors (Brummett et al., 2010). As with the Framingham sample above, the initial DFHS sample included related individuals. We maintained only a single participant from each family cluster.

The public datasets are available upon request to the database of genotypes and phenotypes; dbGaP/database of Genotypes and Phenotypes/National Center for Biotechnology Information, National Library of Medicine, NCBI/NLM; https://www.ncbi.nlm.nih.gov/gap. For the use of Duke datasets, the authors are willing to establish collaboration subject to the approval from the respective Study Committee and Duke IRB.

### Genotyping

The genotyping platform ABI 7900 Taqman system (Applied Biosystems) was used to genotype SNPs in DCS, DFHS, and CHASE; Taqman (Life Technologies) and the QuantiFast Multiplex PCR+ROX kit (Qiagen) were used for ST**RRID**E-PD; Affymetrix Mapping250K (Nsp and Sty) Arrays and Mapping50K (Hind240 and Xba240) Arrays was used for Framingham Cohort; and Affymetrix Genome-Wide Human SNP Array 6.0 was used in MESA, CARDIA, WHI, ARIC, and JHS. We chose the candidate *HTR2C* SNP rs6318 from the genotyping data of these studies. If the SNP was not available in a study, we identified a proxy SNP rs2428722 in high linkage disequilibrium (*R*^2^> = 0.93 in all 1000 Genomes subpopulations) with the *HTR2C* SNP (Singh et al., 2018).

### Phenotypes and Stress Score

For the measurement of depressive symptoms, some form of the Center for Epidemiological Studies Depression Scale [CES-D; (Radloff, 1977)] was available for 6 of the 10 studies used in this analysis. For studies that did not include the CES-D, we used either an alternative measure specifically designed to assess depressive symptoms or a measure that could serve as a suitable proxy given the known to be correlated with formal depression measures. In the CHASE Study, the Beck Depression Inventory and Obvious Depression Scale were available. We converted these two scores to z-scores and calculated the mean of those scores. In the ARIC study, the Maastricht Vital Exhaustion Score (Appels et al., 1987) was used, while in the ST**RRID**E-PD study, the mental health scale (with scoring reversed) from the SF-36 (Ware, 1993) served as the depression measure. All measures were then standardized to z-scores (mean of 0 and standard deviation of 1) within each respective study to create a common depressive symptom variable (Singh et al., 2018). BMI was available in all studies, and was calculated as kg/m^2^.

For the chronic stress measure, out of the 10 studies that we included in the present study only two, MESA (Shivpuri et al., 2012) and JHS (Johnson et al., 2016), had self-rated stress measures. In our prior work on data harmonization (Singh et al., 2018), in the eight studies that lacked a self-rated stress measure we constructed a stress variable using an algorithm (Singh et al., 2015) based on proxy indicators of five stress domains: financial, marital, work, health of spouse, and one’s own health. These domains were based on the chronic burden items from the MESA study (Shivpuri et al., 2012) that were derived from a composite stress measure originally developed in the Study of Women’s Health Across the Nation (Troxel et al., 2003). Briefly, our algorithm (Singh et al., 2015) searched for proxy indicators of each stress domain, scored each proxy item as 1 = stressful, 0 = not stressful. The item scores were then summed resulting in a single score. In some instances, not all indicator domains were available, resulting in varying possible score ranges across studies. In order to harmonize the differently scaled measures, we standardized the score within each study by transforming them to z-scores (mean of zero and a standard deviation of one). For additional details regarding this measure see (Singh et al., 2015, 2018).

### Statistical Analyses

The background characteristics were described using means and standard deviations for continuous variables, and frequency and percentages for the categories. The primary analyses were carried out using random effects models as implemented in SAS Proc Mixed (SAS Institute, Cary, NC, United States), adjusting *a priori* for age. Because Ser23Cys is X-linked, and because two studies were exclusively one race (Jackson Heart, Black; Framingham Offspring Study, White) we estimated separate models for each race and sex combination, and for each phenotype (depressive symptom score and BMI). Preliminary analyses with restricted cubic splines also found a strong non-linear association between age and BMI. An additional quadratic term for age served as a reasonable approximation of the non-linear form in all models. Study source was specified as a random effect (intercept only), and the phenotype of interest (the standardized depressive symptoms score or BMI) as the response variable. The models proceeded by first evaluating a stress by SNP interaction including subordinate main effects in the model. If the interaction term was not statistically significant, we re-estimated a model with only main effects. The fixed portion of the interaction model, then, took the following form:

$${\text{Phenotype}}_{\text{ij}}={\text{β}}_{\text{0j}}+{\text{β}}_{1\text{j}}\text{Age}+{\text{β}}_{2\text{j}}{\text{Age}}^{2}+{\text{β}}_{3\text{j}}\text{SNP}+{\text{β}}_{4\text{j}}\text{Stress}+{\text{β}}_{5\text{j}}SNP*Stress$$

and the random portion was:

$${\text{β}}_{\text{0j}}=\gamma_{00}+{\text{u}}_{0\text{j}}.$$

The first equation represented the fixed effects component of the model, where the phenotype was either depressive symptoms or BMI, β_0_, the model intercept, and each β represented the slope coefficient for each predictor term in the model. The second equation was the random effects component, in which γ represented the grand mean of the phenotype for each study when the predictors had a value of zero (i.e., the mean of the study intercepts); u was the variance of the intercepts for all studies around the overall mean intercept.

Given the relatively large sample size, we also pre-specified the effect sizes that would be considered clinically meaningful. The depression score scale varied considerably across studies and was thus standardized for the analysis. Therefore, the pre-specified effect size was also expressed in terms of a standardized score: we required an effect to be at least 0.5 SDs to be considered clinically significant for the depressive symptom score. BMI, in contrast, was available in the original metric of kg/m^2^ in all studies: we therefore specified the clinical significance threshold in those original units. Based on our prior clinical and investigative experience with BMI, we selected 0.5 kg/m^2^ as the clinical significance criterion.

Age, BMI, depressive symptoms and the stress score were modeled as continuous variables. Prior work with the stress score supports this approach (Singh et al., 2014). Based on an examination of model residuals, the standardized depressive symptom variable was transformed using the square root after adding a constant of 2 to each z-score. For the models with females, the genotype was coded as a 3-level factor: Ser/Ser, Cys/Ser, and Cys/Cys. The major allele homozygotes (Cys/Cys) were used as the reference group. The 2-degree-of-freedom test was used to interpret results involving the genotype. As males have only one copy of a given allele and therefore only two possible genotypes, genotype was coded as binary, Ser/- and Cys/-, with the major allele hemizygotes (Cys/-) as the reference. We used Bonferroni correction for all tests of regression coefficients, applying the same correction to each term in a given model. We based the correction on the number of models. Thus, with 8 separate models (2 phenotypes × 2 sexes × 2 races), the resulting required alpha was 0.006.

We conducted two types of sensitivity analyses. In the first we estimated the same models described above for each study that had ancestry markers available (ARIC, Framingham, MESA, WHI), adding the principal component weights to the model as adjustment variables. In the second sensitivity analysis, we re-estimated the primary random effects models after converting depressive symptom scores and BMI to ranks, rendering the results parallel to a non-parametric analysis. Finally, we used Cochrane’s *Q* statistic and forest plots from the metafor package^1^ in R to assess study heterogeneity for the unstandardized SNP by stress interaction coefficients. Coefficients for the heterogeneity analysis were derived using SAS PROC GLM, estimating a separate model for each study within each race and gender.

## Results

### Descriptive Analyses

Descriptive statistic of the background characteristics are displayed in **Table 1**. The genotype frequencies we observed were consistent with those known for each race and sex (Lappalainen et al., 1995). Similarly, consistent with prior literature, Black women had the greatest BMI, White women the lowest. Black men and women exhibited greater stress scores compared to whites, and women of both races had higher depressive symptom scores compared to men.

**Table 1:** Descriptive statistics by race and sex.

	Black women	Black men	White women	White men	*P*
	**10457**	**2819**	**7419**	**6466**	
rs6318, N (%)					<0.001
Ser/Ser	1327 (12.7)		217 (2.9)		
Cys/Ser	4862 (46.5)		2041 (27.5)		
Cys/Cys	4268 (40.8)		5161 (69.6)		
rs6318 Allele frequency, N (%)					
Ser	7516 (35.9)	1062 (37.7)	2475 (16.7)	1122 (17.4)	
Cys	13398 (64.1)	1757 (62.3)	12363 (83.3)	5344 (82.6)	
Age, mean (SD)	56.7 (11.4)	50.65 (14.59)	54.3 (9.6)	55.09 (9.64)	<0.001
BMI, mean (SD)	30.8 (6.6)	28.12 (5.45)	26.7 (5.5)	27.46 (4.01)	<0.001
Stress Z-Score, mean (SD)	0.18 (1.1)	0.19 (1.06)	-0.08 (0.9)	-0.25 (0.85)	<0.001
Depressive symptoms Z-Score, mean (SD)	0.07 (1.0)	-0.12 (0.90)	0.10 (1.0)	-0.24 (0.88)	<0.001

### Model Results

**FIGURE 1:**
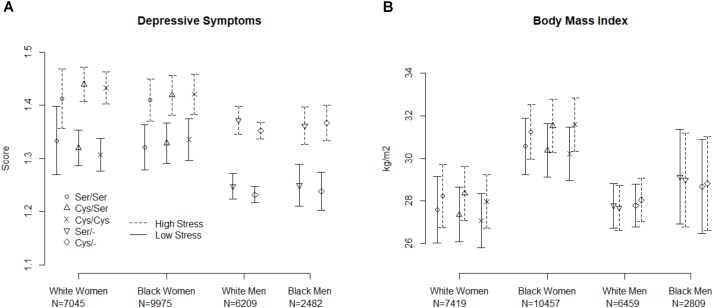
Predicted mean and 95% confidence interval for depressive symptoms **(A)** and body mass index **(B)** by genotype and stress level for males and females. Values were generated from random intercepts model using select values or the stress measure (25th and 75th percentile of the standardized score).

**Figure 1** displays the model-fitted mean and 95% confidence interval of the depressive symptom score (Panel A) and BMI (Panel B) for each sex/race combination. The *p*-values and R-squares for the model terms appear in **Table 2**. Given our adjusted *p*-values criterion, there were no statistically significant genotype by stress interactions for the outcomes of depression or BMI main effect relationships of the rs6318 genotype with either depression or BMI within any race/sex group. Similarly, for all race/sex groups there were no main effect associations between the genotype and depressive symptoms or BMI.

**Table 2:** *P*-values and R-squares for terms in random effects model results.

	Females						Males					
	SNP × Stress		SNP		Stress		SNP × Stress		SNP		Stress	
Depression	*p*	*R*^2^	*p*	*R*^2^	*p*	*R*^2^	*p*	*R*^2^	*p*	*R*^2^	*p*	*R*^2^
White	0.281	0.00016	0.522	0.00019	<0.0001	0.0756	0.810	0.00042	0.086	0.00045	<0.0001	0.0716
Black	0.793	0.00017	0.438	0.00017	<0.0001	0.0642	0.236	4 × 10^-6^	0.849	4 × 10^-6^	<0.0001	0.1082
BMI												
White	0.706	0.0009	0.039	0.0009	<0.0001	0.0160	0.058	0.00050	0.207	0.00026	0.005	0.0012
Black	0.012	0.0252	0.583	0.0001	<0.0001	0.0243	0.214	0.00063	0.242	0.00044	0.778	0.00004

*Models included age and age^2^ as adjustment variables and study as a clustering variable. P-values for main effects are derived from model with main effects only.*

The most pronounced effect involving Ser23Cys was an interaction with stress predicting BMI in Black women. In this case, Ser homozygotes under high stress had lower BMI levels compared to Cys homozygotes under high stress. Conversely, however, Ser homozygotes under low stress had greater BMI compared to Cys homozygotes under low stress. However, the observed differences were below our threshold for clinically important differences, and as noted above, the *p*-values exceeded the pre-specified alpha level of 0.006.

The most consistent result among the models was the main effect for stress on both depressive symptoms and BMI. A strong association was noted in all but one race and sex group, with greater stress predicting more severe depressive symptoms, and higher BMI. The one exception was among Black men, for whom stress was only very weakly related to BMI. Expressed in standardized effect sizes, a one standard deviation increase in the stress score was associated with increases in the depressive symptom score of 0.33 standard deviations in White females, 0.23 in Black females, 0.28 in White males, and 0.29 in Black males. In the parallel model for BMI, a one standard deviation increase in the stress score was associated with a 0.75 standard deviation increase in BMI in White females, 0.95 in Black females, 0.16 in White males, and 0.03 in Black males.

**FIGURE 2:**
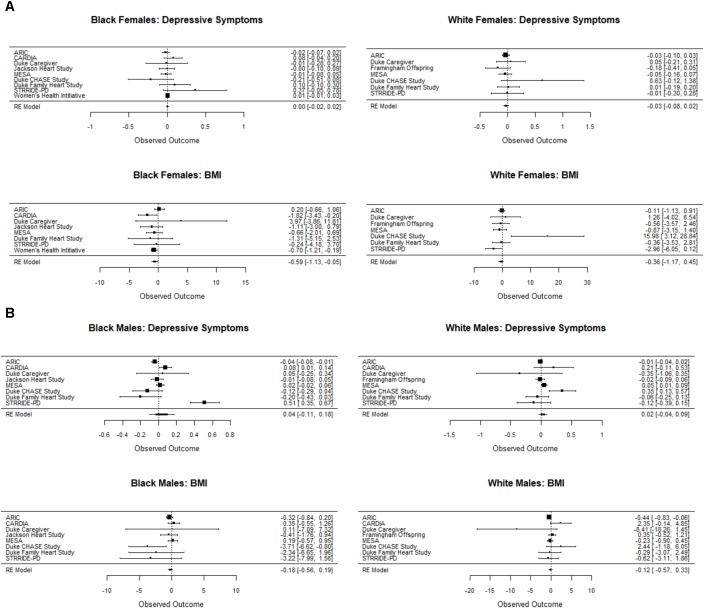
Forest plot showing the unstandardized coefficient for the interaction between Ser/Ser vs. Cys/Cys and the stress measure for each study for the depressive symptom and BMI outcomes for females **(A)** and males **(B)**. Cochrane’s *Q* test for between-study heterogeneity was statistically significant only for White males and Black males on the depressive symptom outcome. Coefficients were generated for each study separately using a general linear model. Results were consistent with tests of a SNP by stress by study interaction term in a random intercepts model. In the study-by-study analysis, data were too sparse in the ST**RRID**E AT/RT dataset to estimate the SNP by stress interaction and thus not included in the heterogeneity analysis.

Sensitivity analyses adjusting for ancestry markers, where available, did not materially alter the results. Additional supplementary analyses using non-parametric tests also were consistent with the parametric results reported above. Further, using the *Q*-test, we found no strong evidence of study heterogeneity with respect to the rs6318 by stress interaction for BMI for women and men of either race (Black women, *p* = 0.39; White women, *p* = 0.14; Black men, *p* = 0.15; White men, *p* = 0.11). The test for heterogeneity was also not significant for depressive symptoms for women of either race (Black women, *p* = 0.34; White women, *p* = 0.50). We did observe heterogeneity for both black males (*p* < 0.0001) and white males (*p* < 0.007) with respect to depressive symptoms. The effects are displayed for each phenotype by race/sex group in the forest plots in **Figure 2**. As a check, we also tested a SNP by stress by study interaction term using the random intercepts model. As would be expected, the results were consistent with the above study-by-study heterogeneity analyses.

## Discussion

The present study suggests that the Cys23Ser rs6318 polymorphism is not associated with depressive symptoms or BMI regardless of the presence or absence of psychosocial stress. The lack of association between Cys23Ser and depressive symptoms or BMI is consonant with at least some prior work: results from a 2017 systematic review and meta-analysis (Gonzalez-Castro et al., 2017) that evaluated the role of the variant Cys23Ser (rs6318) in the pathogenesis of suicidal behavior also found no association. Similarly, a recent comprehensive review and meta-analysis (Gressier et al., 2016) found no association for weight gain when examining the prototypic antipsychotic clozapine. Given the small effect sizes we observed for terms involving this single SNP, it also may be that the effects exist, but were simply too small to detect with our sample sizes given our *a priori* criteria for clinical and statistical significance. It is possible that extending our work to a polygenic model may yield more clinically meaningful effect sizes; multiple SNPs in *HTR2C* may impact the regulation and function of HTR2C, resulting in variable phenotypic expression. For example, one SNP, rs1414334 that is in high LD with rs6318 (*R*^2^ = 0.92 in European-Americans) was associated with metabolic syndrome related to antipsychotic drug use (Mulder et al., 2009; Risselada et al., 2012). Indeed, two of the largest genome-wide association studies on depression have recently reported 44 (Wray et al., 2018) and 17 loci (Howard et al., 2018) significantly related to depression. In the largest GWAS of major depression to date, Wray et al. (2018)reported odds ratios on the order of 1.03–1.04, indicating very small effect sizes and requiring very large samples to detect these SNP effects. These studies did not report analyses of the X-chromosome and thus the relative importance of rs6318 in these large analyses is unknown. Further, our observation of possible differential effects of rs6318 by race and gender adds to the complexity of the genetic model. The effects we report for women, in particular, suggest that it may be worthwhile to continue investigation of Cys/Ser in future studies, and also highlight the potential importance of considering sex when evaluating SNP effects. This observation coupled with the observed genetic architecture for depression makes it plausible that this coding SNP could have effects on brain-related phenotypes that would require much larger sample sizes to detect. The potential for additional functional variants coupled with the negative findings in the harmonized dataset suggest the need for additional genetic analysis of HTR2C. Regardless, this is the largest study of rs6318 to date and thus provides at least some clarity around the inconsistency of the associations.

In contrast to the lack of relationship between rs6318 with depressive symptoms, and BMI, we found consistent and relatively strong associations of chronic stress with both depressive symptoms and BMI. Indeed, with the exception of Black males with respect to BMI, higher chronic stress was associated with higher depressive symptoms scores and higher BMI within *each* race and sex subgroup. While it is generally understood that chronic psychosocial stress is an important risk factor with regard to physical and mental health outcomes (Juster et al., 2010), it has also been suggested that chronic stress may play a key role in racial/ethnic health disparities—through both biological and psychological mechanisms (Jackson et al., 2010). Body weight has been proposed as a potential mediator in these relations (Kim et al., 2009); in related work we have shown that that associations between adiposity and blood pressure vary for Whites and Blacks (Brummett et al., 2012). Although not the primary focus of the current study, we note that while the magnitude of the association between stress and BMI was fairly large in Blacks and Whites, the overall levels of BMI were observed to be higher for Blacks as compared to Whites.

The present study has several limitations. First, as with any meta-analysis, using different measures to represent a single common underlying phenotype, as was the case for our measures of depressive symptoms and psychosocial stress, assumes that the measures are similar enough to adequately represent the same underlying phenotype. Although this assumption is not directly testable using standard psychometric techniques (e.g., common factor analysis) in the data available to us, the depressive symptom measures in each study behaved as expected: average scores for females were consistently higher than those for males in all but one study (Duke Family Heart Study), with White men having the lowest scores across all groups. The content of the psychosocial stress measure also differed across studies. Psychometric analyses conducted on this measure in our prior work (Singh et al., 2015) demonstrated that the various indicators of stress were congeneric, meaning that they were essentially exchangeable. We also observed that the correlation between stress measure and depressive symptoms was positive and similar in magnitude in all but the CARDIA study (Singh et al., 2018). Although the psychosocial stress measure represents structural, objective stressors, we cannot know whether these resulted in the subjective perception of stress. This concern is somewhat allayed given the consistent association with depressive symptoms. In addition, we observed significant heterogeneity across studies for both Black and White males with respect to the SNP by stress interaction and depressive symptoms. Thus, there is less certainty regarding the model estimates in these two groups. Finally, ancestry markers were not available for several of the studies. We therefore were unable to include population stratification adjustment in our primary analyses. We did, however, conduct a sensitivity analysis in which only the studies that did include ancestry markers were evaluated and observed that the pattern of results was essentially unchanged with this adjustment.

In conclusion, the current findings do not support the hypothesis that the Cys23Ser rs6318 polymorphism is related to depressive symptoms or BMI, regardless of the level of psychosocial stress. However, these data do support prior work indicating that chronic stress is significantly related to both depressive symptoms and adiposity.

## Ethics Statement

This study was carried out in accordance with the recommendations of ‘Duke Institutional Review Board’ with written informed consent from all subjects. All subjects gave written informed consent in accordance with the Declaration of Helsinki. The protocol was approved by the ‘Duke Institutional Review Board’.

## Author Contributions

RW, IS, BB, EH, MB, RJ, AS, SS, and WK contributed to the conception and design of the study. BB wrote the initial draft of manuscript. MB, EH, AS, and SS wrote sections of the manuscript. MB performed the statistical analyses. AS, MB, BB, and RJ organized the databases. All authors contributed to the manuscript revision, read, and approved the submitted version.

## Conflict of Interest Statement

The authors declare that the research was conducted in the absence of any commercial or financial relationships that could be construed as a potential conflict of interest.

## Footnotes

1 https://www.r-project.org/
